# Development of a 5-Year Risk Prediction Model for Transition From Prediabetes to Diabetes Using Machine Learning: Retrospective Cohort Study

**DOI:** 10.2196/73190

**Published:** 2025-05-09

**Authors:** Yongsheng Zhang, Hongyu Zhang, Dawei Wang, Na Li, Haoyue Lv, Guang Zhang

**Affiliations:** 1 Department of Health Management The First Affiliated Hospital of Shandong First Medical University & Shandong Provincial Qianfoshan Hospital Jinan China; 2 Shandong Engineering Research Center of Health Management, Shandong Institute of Health Management The First Affiliated Hospital of Shandong First Medical University & Shandong Provincial Qianfoshan Hospital Jinan China; 3 Department of Occupational Health Shizhong District Center for Disease Control and Prevention Jinan China; 4 Postgraduate Department Shandong First Medical University, Shandong Academy of Medical Sciences Jinan China

**Keywords:** Chinese population, CatBoost, SHAP, risk factors, clinical decision support

## Abstract

**Background:**

Diabetes has emerged as a critical global public health crisis. Prediabetes, as the transitional phase with 5%-10% annual progression to diabetes, offers a critical window for intervention. The lack of a 5-year risk prediction model for diabetes progression among Chinese individuals with prediabetes limits clinical decision-making support.

**Objective:**

This study aimed to develop and validate a machine learning–based 5-year risk prediction model of progression from prediabetes to diabetes for the Chinese population and establish an interactive web-based platform to facilitate high-risk patients identifying and early targeted interventions, ultimately reducing diabetes incidence and health care burdens.

**Methods:**

A retrospective cohort study was conducted on 2 prediabetes cohorts from 2 Chinese medical centers (primary cohort: n=6578 and external validation cohort: n=2333) tracking from 2019 to 2024. Participants meeting the American Diabetes Association (ADA) criteria (prediabetes: hemoglobin A1c [HbA1c] level of 5.7%-6.4%; diabetes: HbA1c level of ≥6.5%) were identified. A total of 42 variables (demographics, physical measures, and hematologic biomarkers) were collected using standardized protocols. Patients were split into the training (70%) and test (30%) sets randomly in the primary cohort. Significant predictors were selected on the training set using recursive feature elimination methods, followed by prediction model development using 7 machine learning algorithms (logistic regression, random forest, support vector machine, multilayer perceptron, extreme gradient boosting machine, light gradient boosting machine, and categorical boosting machine [CatBoost]), optimized through grid search and 5-fold cross-validation. Model performance was assessed using the receiver operating characteristic curve, the precision-recall curves, accuracy, sensitivity, and specificity as well as multiple other metrics on both the test set and the external test set.

**Results:**

During the follow-up of 5 years, 2610 (41.6%) participants and 760 (35.2%) participants progressed from prediabetes to diabetes, with mean annual progression rates of 8.34% and 7.04% in the primary cohort and the external cohort, respectively. Using 14 features selected using the recursive feature elimination-logistic algorithm, the CatBoost model achieved optimal performance in the test set and the external test set with an area under the receiver operating characteristic curve of 0.819 and 0.807, respectively. It also showed the best discrimination performance on the accuracy, negative predictive value (NPV), and F1-scores as well as the calibration performances in both the test set and the external test set. Then the Shapley Additive Explanations (SHAP) analysis highlighted the top 6 predictors (FBG, HDL, ALT/AST, BMI, age, and MONO), enabling targeted modification of these risk factors to reduce diabetes incidence.

**Conclusions:**

We developed a 5-year risk prediction model of progression from prediabetes to diabetes for the Chinese population, with the CatBoost model showing the best predictive performance, which could effectively identify individuals at high risk of diabetes.

## Introduction

Diabetes has become a severe global public health concern, affecting 537 million adults aged 20-79 years worldwide and threatening 860 million adults worldwide due to impaired glucose tolerance and impaired fasting glucose, which are commonly known as prediabetes [1]. The burden of diabetes now surpasses the combined global impact of tuberculosis, AIDS, and malaria [2]. In 2021, China had approximately 141 million adults with diabetes and 197 million adults with prediabetes, ranking first globally for both conditions [1]. Prediabetes is typically viewed as a transitional stage of diabetes, which can return to normal or progress to diabetes [3]. Around 5%-10% of patients with prediabetes progress to diabetes every year, and up to 70% could develop diabetes eventually [4,5]. However, with lifestyle intervention and medications, the risk of diabetes in patients with prediabetes can be greatly decreased [6]. Therefore, identifying high-risk individuals among patients with prediabetes and intervening in advance could greatly reduce the incidence and health care burden of diabetes.

Machine learning has been widely used in the field of diabetes and prediabetes, including early diagnosis and risk prediction. Xue et al [7] built four machine learning models to diagnose type 2 diabetes using physical measurements and questionnaire information. Abnoosian et al [8] developed an integrated multiclassifier machine learning model using various data preprocessing techniques and machine learning algorithms to identify patients with 3 conditions: diabetic, prediabetic, and nondiabetic. Hu et al [9] constructed a model to predict the 5-year risk of developing prediabetes in the Chinese population. Schallmoser et al [10] created 2 machine learning models for predicting the risk of microvascular or macrovascular complications within 5 years in patients with prediabetes or diabetes. In terms of the progression from prediabetes to diabetes, Cahn et al [11] constructed a 1-year risk prediction model using 3 datasets, demonstrating superior accuracy (area under the receiver operating characteristic curve [AUC] 0.865-0.925) over logistic regression. Liu et al [12] developed a 1- or 2-year risk prediction model for the Chinese older adults based on extreme gradient boosting machine (XGBoost), demonstrating modest predictive performance (AUC 0.67). Chen et al [13] built a 3-year risk prediction model for the Chinese based on logistic regression using 9 indicators and achieved optimal performance (AUC 0.78). Aoki et al [14] created a 5-year risk prediction model (AUC 0.87) for Americans based on the random forest using 8 laboratory indicators. Although these predictive models for the progression of prediabetes have been developed, Chinese-focused models are limited to short observation periods (≤3 years), and there is currently a lack of a 5-year prediction model specifically tailored to the Chinese population. Prediabetes often undergoes a gradual progression that can last for over 10 years; hence, a long-term risk prediction model would be more meaningful than a short-term prediction model. Therefore, to address this clinical and geographical gap, we aimed to develop and validate a 5-year risk prediction model for the progression from prediabetes to diabetes within the Chinese population, and to establish an interactive web-based platform for easy clinical practice.

## Methods

### Participants

**Figure 1:**
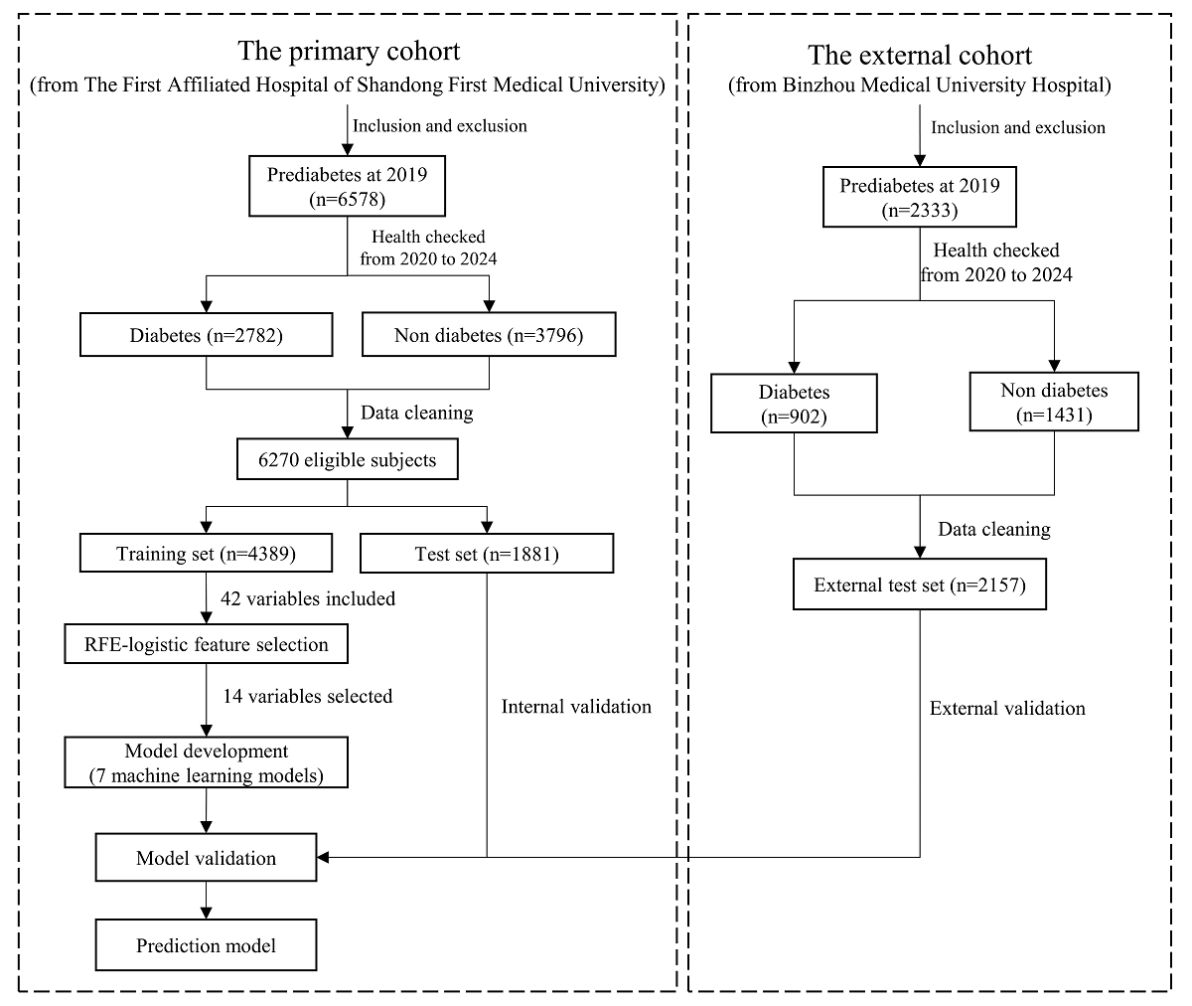
Flowchart of this study. RFE: recursive feature elimination.

This research was a retrospective cohort study that used health checkup data from 2 independent medical centers, namely the First Affiliated Hospital of Shandong First Medical University (the primary cohort) and Binzhou Medical University Hospital (the external cohort). Participants were tracked from 2019 to 2024 over a consecutive 5-year period, with annual assessments conducted at a fixed time each year to evaluate diabetes progression based on the American Diabetes Association (ADA) diagnostic criterion of a hemoglobin A_1c_ (HbA_1c_) level of ≥6.5% [15]. Inclusion criteria required participants to have a confirmed diagnosis of prediabetes (HbA_1c_ level of 5.7%-6.4%) at the 2019 baseline examination. Exclusion criteria ‌included pre-existing diabetes (HbA_1c_ level of ≥6.5% or use of glucose-lowering therapy), history of gestational diabetes, use of medications known to affect glucose metabolism, any previous diagnosis of malignancy, and absence of a second physical examination after the year of 2019. After finalizing study cohort and variable selection, data cleaning was performed by removing participants with any missing values. This complete-case analysis approach is methodologically sound when the proportion of missing data is minimal, as it ensures data integrity while maintaining sufficient statistical power. The study design is illustrated in Figure 1.

### Data Collection

We collected 42 variables in total from the database based on literature review, clinical expertise, and data availability, including demographics, physical measures, and hematologic biomarkers. These variables are gender, age, height, weight, BMI, systolic blood pressure, diastolic blood pressure, white blood cell, neutrophil count, lymphocyte count, monocyte count (MONO), hemoglobin, red blood cell (RBC), platelet count, alanine aminotransferase (ALT), aspartate aminotransferase (AST), ALT to AST ratio (ALT/AST), gamma-glutamyl transferase, alkaline phosphatase, total bilirubin, direct bilirubin, indirect bilirubin, total protein, albumin, globulin, albumin to globulin ratio (albumin/globulin), triglycerides, total cholesterol (TC), high-density lipoprotein cholesterol (HDL), low-density lipoprotein cholesterol (LDL), HDL to TC ratio (HDL/TC), blood urea nitrogen (BUN), creatinine, uric acid, estimated glomerular filtration rate, BUN to creatinine ratio (BUN/creatinine), fasting blood glucose (FBG), hematocrit, mean corpuscular hemoglobin, triglyceride-glucose, monocyte to high-density lipoprotein cholesterol ratio, and neutrophil to high-density lipoprotein cholesterol ratio.

Physical and laboratory examinations are carried out by trained medical staff in accordance with uniform standards. Height and weight measurements require participants to remove heavy clothing and take off shoes. The fully automatic electronic sphygmomanometer was used to measure the participants’ blood pressure in the right upper arm after sitting still and resting for 10 min. The measurement of blood pressure was made twice, each time with an interval of 1-2 minutes, and the average of the 2 readings was taken and recorded. Blood samples were collected from the antecubital vein after an 8- to 10-hour fast and were promptly sent to the clinical laboratory for processing. BMI was calculated as weight (kg) divided by the square of height (m²), and triglyceride-glucose was calculated as Ln (fasting triglycerides [mg/dL] × fasting glucose [mg/dL]/2).

### Statistical Analysis

Baseline characteristics were analyzed using R version 4.3.1 (R Foundation for Statistical Computing). Quantitative data were analyzed using Student *t* test or nonparametric Wilcoxon signed-rank test, while qualitative data were analyzed using chi-square test or Fisher exact test. Statistical significance thresholds were set at *P*<.05 for all comparative analyses.

Model training and validation were conducted using the scikit-learn package (version 1.4.0) in Python (version 3.11.5). Initially, the primary cohort was randomly divided into a training set (comprising 70% of the participants) and a test set (comprising the remaining 30%) [16]. To ensure better model performance and reduce redundant features, recursive feature elimination (RFE) was used on the training set for feature selection with the logistic regression model as the base model [15,17]. Subsequently, 7 machine learning algorithms were selected, namely logistic regression, random forest, support vector machine, multilayer perceptron, XGBoost, light gradient boosting machine, and categorical boosting machine (CatBoost) to construct the prediction models. These models were trained and optimized on the training set, with the optimal parameters determined using grid search and 5-fold cross-validation. The next step involved validating the models’ discrimination and calibration ability using the test set of the primary cohort and the external cohort. The discrimination ability of different models was compared using AUC and the area under the precision-recall (PR) curve, along with accuracy, sensitivity, specificity, positive predictive value, NPV, and *F*_1_-score. The DeLong test was used for comparing AUC differences between models‌. The calibration ability of different models was compared using the calibration curves to assess the consistency between predicted and observed values. The Shapley Additive Explanations (SHAP) method was used to analyze variable importance for the best-performing machine learning model. Finally, decision curve analysis was used to evaluate the clinical application values of each machine learning model, and an interactive web page for easy clinical use was developed using the Python Gradio framework.

### Ethical Considerations

This study followed the principles of the Declaration of Helsinki and was approved by the Ethics Committee of the First Affiliated Hospital of Shandong First Medical University (2024S657), and the informed consent was waived off by the review boards due to the retrospective nature of this research. All the images and tables presented in the manuscript and supplementary materials were anonymized in accordance with ethical standards, ensuring no personally identifiable information could be discerned.

## Results

### Baseline Characteristics

This study enrolled 6270 eligible participants in the primary cohort and 2157 in the external cohort. Over a 5-year period, 2610 participants (41.6%) in the primary cohort and 760 (35.2%) in the external cohort progressed from prediabetes to diabetes, with mean annual progression rates of 8.33% and 7.04%. The characteristics of the primary and external cohorts are shown in Table S1 in Multimedia Appendix 1. Over the 5-year follow-up period, 510 participants were lost to follow-up in the primary cohort. A comparative analysis of baseline characteristics between the lost to follow-up group (n=510) and the completed follow-up group (n=6270) revealed no statistically significant differences in any observed variables (*P*>.05) as shown in Table S2 in Multimedia Appendix 1. Given the lost-to-follow-up–rate of 7.5% (510/6780) and 5.2% (127/2430) in the primary and the external cohort respectively, the demonstrated baseline equivalence between groups, we conclude that the cases lost to follow-up are unlikely to compromise the validity of the analytical outcomes.

### Model Construction and Validation

**Figure 2:**
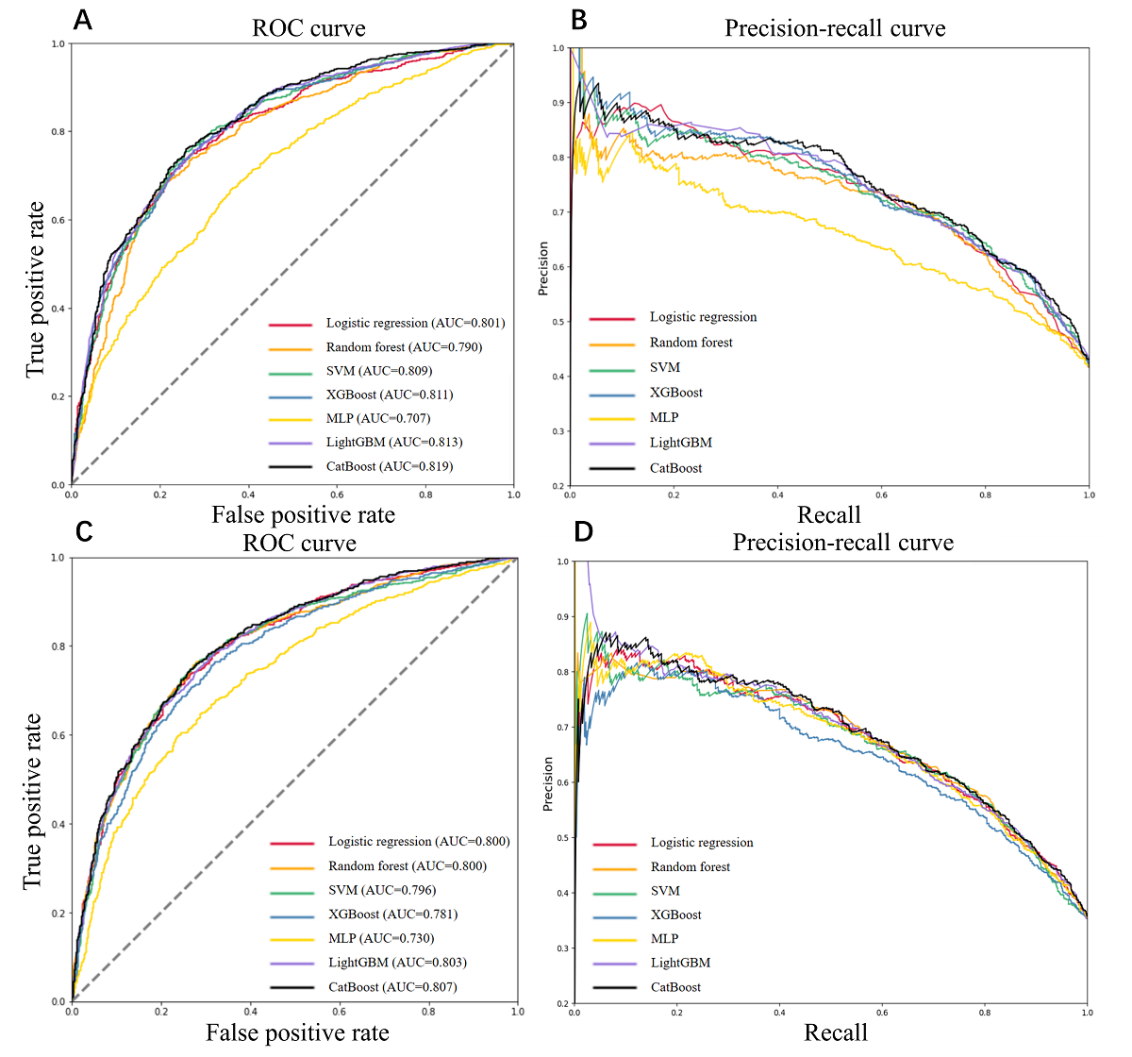
Receiver operating characteristic curve and precision-recall curve curves of the 7 models on the test set and the external test set. Plots A and B depict the model performances on the test set, and plots C and D present the model performances on the external test set. CatBoost: categorical boosting; LightGBM: light gradient boosting machine; MLP: multiplayer perceptron; ROC: receiver operating characteristic; SVM: support vector machine; XGBoost: extreme gradient boosting machine.

The RFE-logistic feature selection algorithm finally screened 14 out of 42 health checkup variables using the training set of the primary cohort, which were hematocrit, hemoglobin, RBC, MONO, FBG, HDL/TC, LDL, HDL, creatinine, ALT/AST, age, height, weight, and BMI. As illustrated in Figure S1 in Multimedia Appendix 1, the RFE-logistic cross-validation analysis demonstrated a plateau in AUC ‌when reaching‌ 14 features, ‌with no significant improvement observed‌ when incorporating more features. As shown in Table S3 in Multimedia Appendix 1, the RFE ranking system eliminated lower-priority variables through iterative cross-validation (where rank=1 denotes retained features, and higher ranks reflect earlier elimination during the feature selection process‌). The discrimination performances of the 7 machine learning models on the test set and the external test set were shown in Figure 2 and Table 1, in which the ROC curves indicated that the CatBoost model had the best performance on both of the test sets (AUC 0.819) and the external test set (AUC 0.807), and the DeLong test revealed that the differences in AUC values for the models on 2 cohorts were statistically significant (*P*<.05). The PR curve analysis further confirmed the superior discriminative performance of the CatBoost model, demonstrating significant advantages on both sets. In addition, the CatBoost model showed the best accuracy (74.6%), sensitivity (0.648), NPV (0.765), *F*_1_-score (0.68) on the test set, and the best accuracy (75.9%), NPV (0.787), and *F*_1_-score (0.626) on the external test set. The confusion matrix for the 7 machine learning models on both sets is shown in Figure S2 in Multimedia Appendix 1.

**Figure 3:**
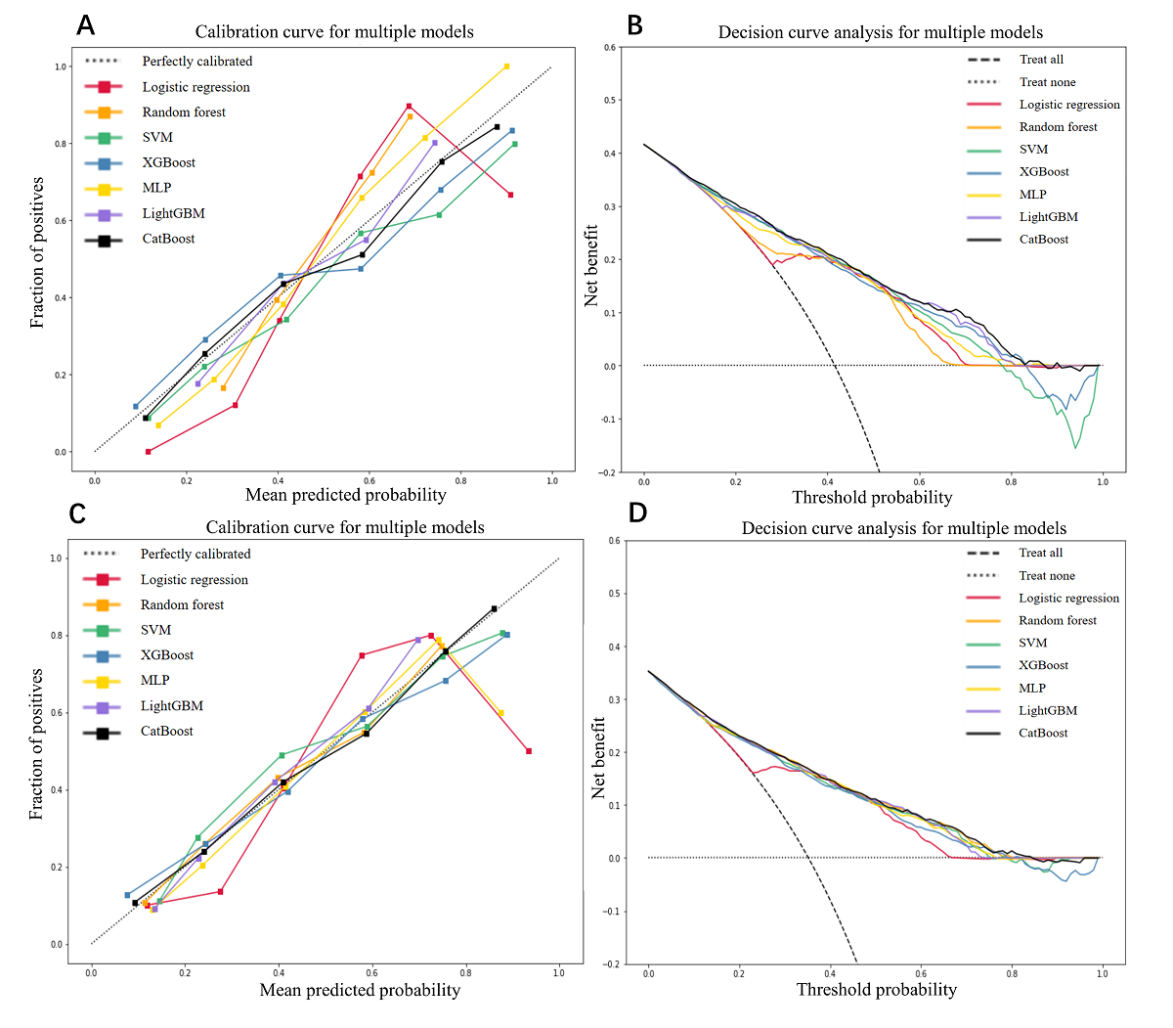
The calibration curves and clinical decision curves of the 7 models on the test set and the external test set. Plots A and B depict the model performances on the test set, and plots C and D present the model performances on the external test set. CatBoost: categorical boosting; LightGBM: light gradient boosting machine; MLP: multiplayer perceptron; SVM: support vector machine; XGBoost: extreme gradient boosting machine.

Meanwhile, the calibration performances of the 7 models on the test set and the external test set were evaluated using calibration curves as shown in plots A and C in Figure 3, in which the black curve corresponding to the CatBoost model had been distributed closest to the dashed line in the middle, indicating the best calibration ability in both sets. The clinical application value of the 7 models on the test set and the external test set were evaluated using decision curves as shown in plot B and D in Figure 3, in which the area under the curve of the CatBoost model reached the maximum, and when the threshold probability was less than around 0.80, all the clinical decisions made would be beneficial to the patients.

**Table 1:** The metrics of model performance on the test set and the external test set.

Models			Accuracy		Sensitivity		Specificity		PPV^a^		NPV^b^		*F*_1_-score
**The test set in the primary cohort**													
	Logistic regression	0.739		0.577		0.854		0.738		0.739		0.648	
	Random forest	0.741		0.592		0.846		0.732		0.745		0.655	
	SVM^c^	0.677		0.277		0.961		0.834		0.651		0.417	
	XGBoost^d^	0.74		0.647		0.806		0.704		0.762		0.674	
	MLP^e^	0.665		0.367		0.877		0.68		0.66		0.477	
	LightGBM^f^	0.744		0.61		0.838		0.729		0.751		0.664	
	CatBoost^g^	0.746		0.648		0.816		0.715		0.765		0.68	
**The external test set**													
	Logistic regression	0.749		0.45		0.913		0.737		0.753		0.559	
	Random forest	0.755		0.578		0.852		0.68		0.786		0.624	
	SVM	0.75		0.468		0.904		0.727		0.758		0.57	
	XGBoost	0.74		0.554		0.842		0.656		0.776		0.601	
	MLP	0.717		0.499		0.836		0.623		0.754		0.554	
	LightGBM	0.747		0.424		0.923		0.75		0.747		0.542	
	CatBoost	0.759		0.572		0.86		0.69		0.787		0.626	

^a^PPV: positive predictive value.

^b^NPV: negative predictive value.

^c^SVM: support vector machine.

^d^XGBoost: extreme gradient boosting.

^e^MLP: multiplayer perceptron.

^f^LightGBM: light gradient boosting machine.

^g^CatBoost: categorical boosting.

### Feature Importance

**Figure 4:**
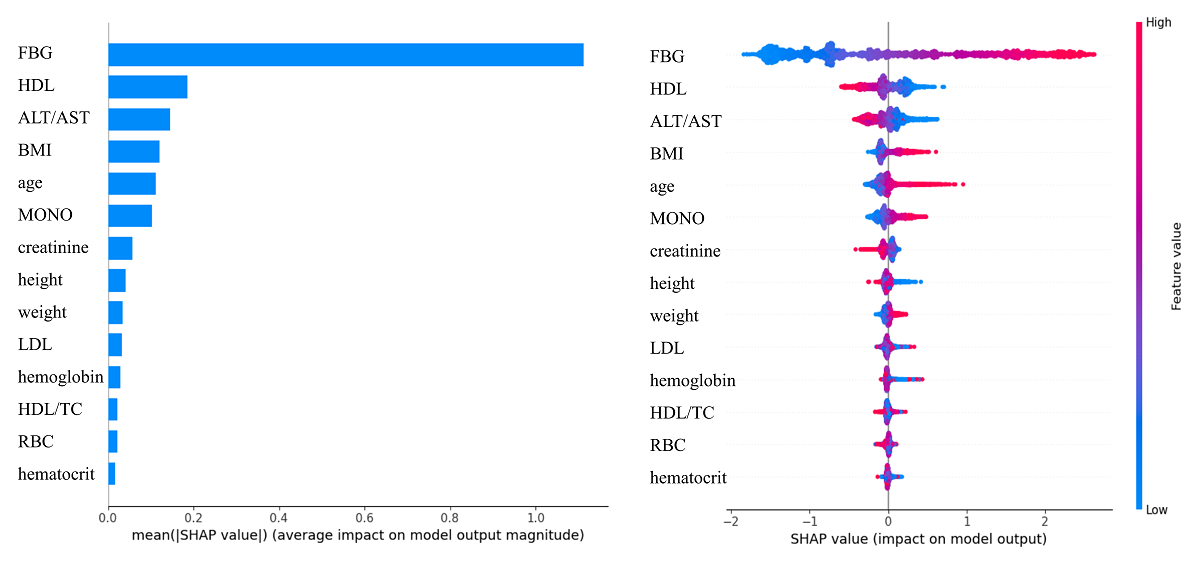
SHapley Additive exPlanations feature importance and summary plot of the CatBoost model. ALT: alanine aminotransferase; AST: aspartate aminotransferase; FBG: fasting blood glucose; HDL: high-density lipoprotein cholesterol; LDL: low-density lipoprotein cholesterol; MONO: monocyte count; RBC: red blood cell.

**Figure 5:**
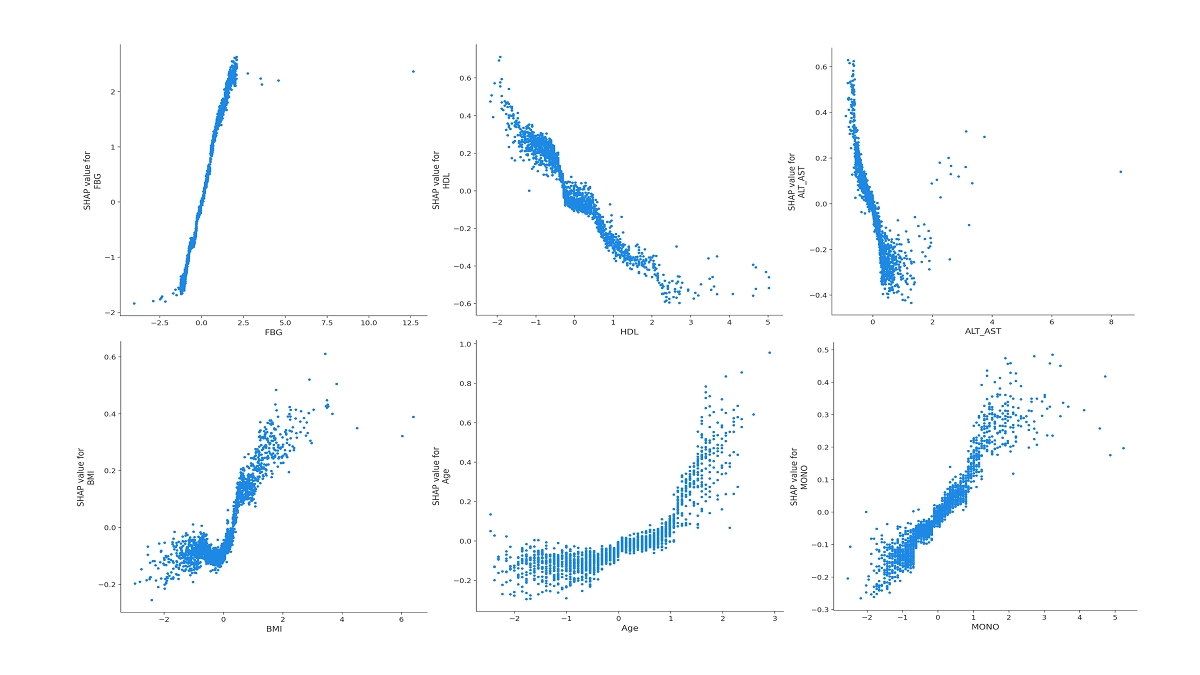
The Shapley Additive Explanations dependency plots for the top 6 features. ALT: alanine aminotransferase; AST: aspartate aminotransferase; FBG: fasting blood glucose; HDL: high-density lipoprotein cholesterol; MONO: monocyte count.

The SHAP analysis of the best-performing CatBoost model is shown in Figure 4. The top 6 variables contributing most to the model were FBG, HDL, ALT/AST, BMI, age, and MONO, where HDL and ALT/AST were negatively correlated with the outcome, that is, protective against progression to diabetes, and the rest were positively correlated and risky for progression to diabetes. Baseline FBG was the most important factor affecting the CatBoost model. In addition, the SHAP dependency plots for the 6 features were shown in Figure 5, which showed the correspondence between the change in the value of variables and the change of importance to the CatBoost model indicated by SHAP values. Finally, we constructed the user-friendly interactive web interface of the prediction model for progression from prediabetes to diabetes as shown in Figure S3 in Multimedia Appendix 1.

## Discussion

### Principal Findings

In this retrospective cohort study, we used 7 machine learning algorithms to build and evaluate 5-year risk prediction models for the progression from prediabetes to diabetes in 2 different cohorts. Among them, the CatBoost model showed the best performance in terms of discrimination and calibration. The CatBoost model achieved the highest AUC values in both the test set (‌0.819‌) and external validation set (‌0.807‌), outperforming all other models. This indicates its exceptional ability to distinguish individuals who will progress to diabetes from those who will not. The model’s AUC values reflect good discrimination for clinical use (AUC ≥0.8 is often considered strong in risk prediction), placing it in the mid-to-high range of published studies (AUC 0.67-0.87) and approaching top-tier models [11-14]. The stability of AUC across internal and external test sets (‌<2% difference‌) suggests that the 14 features selected by RFE-logistic capture biologically and clinically generalizable signals, rather than overfitting to cohort-specific noise. Compared with the traditional logistic regression model, the CatBoost model’s improvement‌ in AUC highlights the value of ensemble algorithms in modeling complex interactions between metabolic, hematological, and anthropometric variables.

Among the 7 evaluated models, the CatBoost algorithm also demonstrated superior performance in sensitivity, NPV, and *F*_1_-score‌. In disease screening and risk assessment contexts, high sensitivity holds particular clinical significance as missed diagnoses of high-risk patients may lead to severe health consequences. Our study also took into consideration the cost-benefit analysis of false-positive cases, systematically balancing the economic and psychological burdens against diagnostic sensitivity through optimized thresholds. The CatBoost model exhibited exceptional capability in low-risk population exclusion, achieving NPV of 76.5% and 78.7% in 2 cohorts. This performance positions it as an ideal solution for resource-constrained health care settings, enabling efficient allocation of medical resources to medium- and high-risk subgroups.

The CatBoost model dominated the space of the precision-recall curve, achieving the highest precision at different recall levels. For example, at a recall of 0.648 in the test set, the model maintained a highest precision of ‌0.715‌. This superiority implies that CatBoost reduces ‌unnecessary interventions‌; for every 100 patients flagged as “high-risk” by CatBoost, 71.5% would truly progress to diabetes. In resource-constrained settings, this precision directly translates to cost savings and reduced patient anxiety. The decision curves further validated the clinical value of the CatBoost model. When the threshold probability for intervention was below 80%, the model provided a net benefit over “treat all” or “treat none” strategies, which means the model remains clinically beneficial across a wide range of risk thresholds, accommodating varying risk tolerance among patients and health care systems.

SHAP analysis of the feature importance identified FBG, HDL, ALT/AST, BMI, age, and MONO as the top 6 variables contributing the most to the CatBoost model, and an interactive web interface for this model was constructed. This interface provided caregivers with an easy tool to assess the 5-year risk of progression to diabetes in patients with prediabetes. It can be integrated into the health information system in China, which could streamline risk assessment, allowing automated alerts for high-risk prediabetic patients during routine health check-ups. By stratifying patients into risk categories, clinicians can prioritize high-risk individuals for intensive monitoring or early lifestyle intervention, such as dietary changes, physical activity plans, or pharmacological approaches in alignment with current clinical guidelines. The web interface bridges the gap between predictive analytics and clinical decision-making, advocating for a paradigm shift toward data-driven, individualized prevention strategies, emphasizing early intervention in high-risk groups to curb diabetes progression.

### Comparisons With Previous Work

The application of machine learning techniques has made significant progress in the field of diabetes, opening up new possibilities for early diagnosis and prevention. CatBoost is an advanced machine learning algorithm designed for gradient boosting with decision trees, applicable to a variety of tasks including classification, regression, and ranking. It leverages techniques such as ordered boosting, stochastic permutations, and gradient-driven optimization to deliver superior results [18-20]. Shojaee et al [18] developed a fasting blood glucose status prediction model using the CatBoost algorithm based on 3376 adults older than 30 years at 16 comprehensive health service centers in Tehran, Iran, which demonstrated good performance with 0.737 AUC. Shiren et al [19] developed a cutting-edge, interpretable risk assessment model for patients with diabetes. The CatBoost algorithm significantly outperformed the other methods in terms of AUC, delivering an impressive average AUC of 90.47% across 4 diabetes-related complications, namely coronary heart disease, diabetic nephropathy, diabetic retinopathy, and nonalcoholic fatty liver disease. Qiu et al [20] constructed a model capable of forecasting the likelihood of cancer in individuals with type 2 diabetes by using tumor biomarkers from a dataset of 5198 patients. The CatBoost model yielded a favorable AUC of 0.852, which might be helpful for early cancer detection and prevention. While CatBoost offers notable advantages, it also has drawbacks. It exhibits higher computational demands compared with lightweight frameworks like light gradient boosting machine (LightGBM), particularly with large datasets. ‌Its performance is sensitive to hyperparameter tuning, demanding significant computational resources and time.‌ In addition, the model’s complexity makes interpretation challenging without external tools like SHAP for transparency.

The variables selected from RFE-logistic algorithm have been proven to be relevant to diabetes in different ways. Changes in hematocrit can alter red blood cell ratios, affecting HbA_1c_ test results and causing overestimation or underestimation of true blood glucose levels in type 2 diabetes [21]. HbA_1c_ had significant correlations with RBC and hemoglobin because chronic hyperglycemia can exacerbate inflammation and alter hematological parameters in diabetic patients [22]. HDL is a protective factor against diabetes, while lower levels of HDL are associated with increased insulin resistance and elevated cardiovascular risk ‌[23]. ALT, TC, triglycerides, HDL, and LDL are clinically significant biomarkers associated with diabetes, which serve as sensitive indicators of insulin resistance and metabolic syndrome [24]. ‌Studies also demonstrate BMI is a modifiable risk factor for diabetes and its macrovascular and microvascular complications. There are positive correlations between BMI and key glycemic control parameters, FBG and HbA_1c_, highlighting its role in β-cell dysfunction and systemic insulin resistance ‌[25]. The results of the feature importance analysis are consistent with the other diabetes studies, where FPG was the most important factor contributing to diabetes risk. As the core diagnostic criterion for diabetes, FBG directly reflects the pathological mechanisms of insulin resistance and β-cell dysfunction‌. Even when FBG levels remain below the diagnostic threshold for diabetes, persistent elevation can accelerate β-cell functional failure through glucotoxic effects‌. In addition, age, BMI, HDL as well as ALT/AST played significant roles in influencing the risk of diabetes [11-14,26-28]. Aging directly contributes to the decline of pancreatic β-cell function and reduced insulin sensitivity‌. With aging, diminished hepatic gluconeogenesis regulation and impaired glucose uptake efficiency in muscle tissues progressively destabilize fasting glucose homeostasis‌, which might underlie the critical transition from prediabetes to diabetes‌. We also found that MONO was an important factor contributing to diabetes risk. Monocytes release a variety of inflammatory mediators and participate in the inflammatory and injury processes in our body, and may prompt the progression from prediabetes to diabetes [29,30].

### Limitations

To the best of our knowledge, this study is the first to develop a 5-year risk prediction model for the progression from prediabetes to diabetes based on the Chinese population. China faces a heavy disease and economic burden due to diabetes and its complications, and identifying high-risk populations among patients with prediabetes could reduce the health care burden of diabetes by implementing lifestyle and pharmacological interventions [31-33]. There are some limitations of our study. First, due to the retrospective nature of this study, the absence of some risk factors, such as smoking, drinking, and the family history of diabetes may introduce residual confounding, while the lack of longitudinal tracking of some variables during follow-up‌ could affect the predictive validity. Second, out of operational practicality, we take HbA_1c_ as the sole diagnostic criterion for diabetes in this study. While HbA_1c_ demonstrates superior accuracy in reflecting long-term glycemic levels [34-36], the exclusion of complementary assessments, such as FBG, oral glucose tolerance test, and clinical symptom evaluation, may introduce diagnostic bias, which might reduce the generalizability of our prediction model. Third, the absence of external validation in populations with distinct genetic backgrounds may limit extrapolation to other ethnic groups. In the future, we will establish prospective cohorts to include more risk factors, diagnostic variables, and implement longitudinal monitoring‌. In addition, we will validate our model across diverse ethnic and geographic populations through multi-center collaborations to enhance the generalizability.

### Conclusions

In conclusion, we constructed and evaluated seven 5-year risk prediction models for the progression from prediabetes to diabetes using machine learning algorithms based on the Chinese health checkup cohorts. The CatBoost model demonstrated the best performance, suggesting its potential as an effective tool in the field of diabetes prevention and management. We can embed the model in electronic health records to flag high-risk individuals during annual checkups, enable targeted allocation of glucose monitoring resources, and facilitate early lifestyle modification to these predicted high-risk patients to reduce diabetes incidence and health care burdens.

## Appendix

Multimedia Appendix 1 Additional material.

## Abbreviations

ADA: American Diabetes Association
ALT: alanine aminotransferase
AST: aspartate aminotransferase
AUC: the area under the receiver operating characteristic curve
BUN: blood urea nitrogen
CatBoost: categorical boosting machine
FBG: fasting blood glucose
HbA1c: hemoglobin A1c
HDL: high-density lipoprotein cholesterol
LDL: low-density lipoprotein cholesterol
LightGBM: light gradient boosting machine
MONO: monocyte count
NPV: negative predictive value
PR: precision-recall
RBC: red blood cell
RFE: recursive feature elimination
ROC: receiver operating characteristic curve
SHAP: Shapley Additive Explanations
TC: total cholesterol
XGBoost: extreme gradient boosting

## Data Availability

The datasets generated or analyzed during this study are available from the corresponding author on reasonable request.
