# Supplementary material for: Development of a 5-Year Risk Prediction Model for Transition From Prediabetes to Diabetes Using Machine Learning: Retrospective Cohort Study
Source: J Med Internet Res. 2025 May 9;27:e73190. doi: 10.2196/73190 (PMC12102623; doi:10.2196/73190)
Supplement: Multimedia Appendix 1 [file jmir_v27i1e73190_app1.docx]

**Table S1.** Baseline Characteristics of the patients with prediabetes in the primary and external cohorts.

| **the Primary Cohort**  **(from the First Affiliated Hospital of Shandong First Medical University)** | | | | | **the External Cohort**  **(from Binzhou Medical University Hospital)** | | | |
| --- | --- | --- | --- | --- | --- | --- | --- | --- |
| **Variables** | **Overall**,  N = 6,270*^1^* | **Non-Diabetes**,  N = 3,660*^1^* | **Diabetes**,  N = 2,610*^1^* | **p-value***^2^* | **Overall**, N = 2,157^1^ | **Non-Diabetes**, N = 1,397^1^ | **Diabetes**, N = 760^1^ | **p-value**^2^ |
| Gender |  |  |  | <0.001 |  |  |  | <0.001 |
| Female | 1,929 (31%) | 1,245 (34%) | 684 (26%) |  | 646 (30%) | 455 (33%) | 191 (25%) |  |
| Male | 4,341 (69%) | 2,415 (66%) | 1,926 (74%) |  | 1,511 (70%) | 942 (67%) | 569 (75%) |  |
| Age | 56 (47, 65) | 56 (47, 64) | 57 (48, 67) | <0.001 | 56 (47, 65) | 56 (46, 64) | 57 (49, 67) | <0.001 |
| Height | 170 (164, 175) | 170 (163, 175) | 170 (164, 175) | 0.10 | 170 (164, 175) | 170 (163, 175) | 170 (164, 175) | 0.6 |
| Weight | 74 (66, 83) | 73 (65, 81) | 76 (68, 85) | <0.001 | 74 (66, 83) | 74 (66, 82) | 76 (67, 85) | <0.001 |
| BMI | 26.0 (24.0, 28.1) | 25.7 (23.7, 27.6) | 26.4 (24.3, 28.7) | <0.001 | 26.0 (24.1, 28.1) | 25.8 (23.8, 27.6) | 26.4 (24.4, 28.7) | <0.001 |
| SBP | 136 (123, 150) | 134 (122, 148) | 138 (125, 151) | <0.001 | 136 (124, 150) | 134 (123, 148) | 138 (126, 153) | <0.001 |
| DBP | 81 (74, 90) | 81 (74, 89) | 82 (74, 90) | 0.069 | 81 (73, 90) | 81 (73, 89) | 83 (74, 91) | 0.024 |
| ALT | 19 (14, 27) | 18 (13, 26) | 20 (14, 30) | <0.001 | 19 (14, 27) | 19 (14, 27) | 20 (14, 30) | <0.001 |
| AST | 20 (17, 23) | 20 (17, 23) | 20 (16, 24) | 0.7 | 20 (17, 24) | 20 (17, 23) | 20 (17, 24) | 0.3 |
| ALT/AST | 1.01 (0.80, 1.29) | 1.05 (0.83, 1.34) | 0.96 (0.75, 1.22) | <0.001 | 1.01 (0.80, 1.28) | 1.04 (0.82, 1.32) | 0.96 (0.76, 1.21) | <0.001 |
| GGT | 26 (18, 39) | 25 (17, 37) | 28 (19, 42) | <0.001 | 26 (18, 39) | 25 (18, 38) | 28 (18, 44) | <0.001 |
| ALP | 65 (55, 76) | 64 (55, 76) | 65 (56, 77) | 0.002 | 65 (55, 77) | 64 (55, 76) | 65 (55, 77) | 0.5 |
| TBIL | 11.6 (9.1, 15.1) | 11.5 (8.9, 14.9) | 11.8 (9.2, 15.2) | 0.038 | 11.7 (9.0, 15.2) | 11.7 (9.0, 15.1) | 11.8 (9.2, 15.3) | 0.6 |
| DBIL | 4.30 (3.40, 5.40) | 4.20 (3.40, 5.30) | 4.30 (3.50, 5.50) | 0.010 | 4.30 (3.50, 5.40) | 4.30 (3.40, 5.40) | 4.35 (3.50, 5.50) | 0.2 |
| IBIL | 7.3 (5.4, 9.8) | 7.2 (5.4, 9.8) | 7.3 (5.5, 9.9) | 0.10 | 7.3 (5.4, 9.9) | 7.3 (5.3, 9.9) | 7.3 (5.5, 9.9) | 0.9 |
| TP | 74.6 (72.0, 77.2) | 74.8 (72.2, 77.4) | 74.3 (71.7, 77.0) | <0.001 | 74.6 (72.1, 77.3) | 74.7 (72.2, 77.4) | 74.3 (72.0, 77.2) | 0.15 |
| ALB | 47.80 (46.10, 49.50) | 47.90 (46.20, 49.60) | 47.60 (46.00, 49.40) | <0.001 | 47.90 (46.20, 49.50) | 47.90 (46.30, 49.50) | 47.70 (46.10, 49.50) | 0.2 |
| GLB | 26.7 (24.6, 28.9) | 26.8 (24.7, 29.0) | 26.6 (24.4, 28.8) | 0.006 | 26.7 (24.5, 29.0) | 26.8 (24.6, 28.9) | 26.6 (24.4, 29.0) | 0.7 |
| ALB/GLB | 1.79 (1.63, 1.97) | 1.79 (1.63, 1.96) | 1.80 (1.63, 1.98) | 0.2 | 1.80 (1.63, 1.97) | 1.79 (1.63, 1.97) | 1.80 (1.63, 1.98) | >0.9 |
| BUN | 4.90 (4.20, 5.70) | 4.90 (4.20, 5.70) | 4.90 (4.20, 5.90) | <0.001 | 4.90 (4.20, 5.70) | 4.90 (4.20, 5.70) | 5.00 (4.25, 5.90) | 0.008 |
| Cr | 75 (65, 84) | 75 (65, 84) | 75 (66, 84) | 0.2 | 75 (65, 84) | 75 (65, 84) | 75 (66, 84) | 0.4 |
| UA | 344 (290, 400) | 340 (285, 397) | 349 (298, 403) | <0.001 | 345 (293, 402) | 340 (288, 400) | 355 (302, 405) | 0.002 |
| EGFR | 99 (89, 108) | 99 (89, 107) | 98 (88, 108) | 0.2 | 98 (89, 108) | 99 (89, 108) | 98 (87, 108) | 0.070 |
| BUN/Cr | 0.066 (0.056, 0.078) | 0.065 (0.056, 0.078) | 0.067 (0.056, 0.079) | 0.012 | 0.066 (0.056, 0.078) | 0.065 (0.056, 0.078) | 0.067 (0.056, 0.080) | 0.039 |
| TG | 1.44 (1.03, 2.07) | 1.39 (0.99, 1.98) | 1.52 (1.09, 2.17) | <0.001 | 1.44 (1.04, 2.09) | 1.42 (1.02, 2.02) | 1.52 (1.08, 2.18) | 0.002 |
| TC | 4.82 (4.19, 5.47) | 4.86 (4.24, 5.49) | 4.77 (4.14, 5.44) | <0.001 | 4.83 (4.19, 5.48) | 4.85 (4.21, 5.47) | 4.77 (4.15, 5.51) | 0.3 |
| HDL | 1.16 (1.02, 1.34) | 1.19 (1.04, 1.39) | 1.12 (0.99, 1.28) | <0.001 | 1.16 (1.03, 1.34) | 1.19 (1.04, 1.38) | 1.13 (1.01, 1.30) | <0.001 |
| LDL | 2.81 (2.29, 3.32) | 2.83 (2.31, 3.33) | 2.80 (2.26, 3.30) | 0.047 | 2.81 (2.29, 3.31) | 2.82 (2.31, 3.30) | 2.81 (2.27, 3.32) | 0.7 |
| HDL/TC | 0.24 (0.21, 0.29) | 0.25 (0.21, 0.29) | 0.24 (0.21, 0.28) | <0.001 | 0.24 (0.21, 0.29) | 0.25 (0.21, 0.29) | 0.24 (0.21, 0.28) | <0.001 |
| FBG | 6.04 (5.78, 6.43) | 5.87 (5.71, 6.10) | 6.40 (6.06, 6.69) | <0.001 | 6.01 (5.77, 6.40) | 5.87 (5.71, 6.12) | 6.43 (6.07, 6.69) | <0.001 |
| WBC | 6.22 (5.35, 7.26) | 6.10 (5.23, 7.12) | 6.41 (5.55, 7.47) | <0.001 | 6.23 (5.38, 7.24) | 6.12 (5.31, 7.19) | 6.38 (5.57, 7.37) | <0.001 |
| NEUT | 3.41 (2.80, 4.15) | 3.32 (2.72, 4.02) | 3.51 (2.92, 4.27) | <0.001 | 3.42 (2.83, 4.13) | 3.35 (2.76, 4.02) | 3.52 (2.97, 4.25) | <0.001 |
| LYM | 2.11 (1.74, 2.55) | 2.08 (1.72, 2.52) | 2.14 (1.77, 2.60) | <0.001 | 2.11 (1.74, 2.54) | 2.09 (1.74, 2.52) | 2.14 (1.74, 2.55) | 0.2 |
| MONO | 0.45 (0.37, 0.54) | 0.44 (0.36, 0.52) | 0.47 (0.39, 0.56) | <0.001 | 0.45 (0.37, 0.53) | 0.44 (0.36, 0.52) | 0.46 (0.39, 0.55) | <0.001 |
| RBC | 151 (140, 160) | 150 (140, 160) | 152 (141, 160) | 0.006 | 152 (141, 160) | 151 (141, 160) | 152 (141, 160) | 0.2 |
| HGB | 4.90 (4.59, 5.20) | 4.88 (4.58, 5.18) | 4.93 (4.61, 5.22) | 0.002 | 4.91 (4.60, 5.20) | 4.90 (4.59, 5.19) | 4.94 (4.63, 5.22) | 0.2 |
| HCT | 0.45 (0.43, 0.48) | 0.45 (0.43, 0.48) | 0.46 (0.43, 0.48) | 0.050 | 0.46 (0.43, 0.48) | 0.46 (0.43, 0.48) | 0.46 (0.43, 0.48) | 0.7 |
| MCH | 30.70 (29.80, 31.70) | 30.70 (29.80, 31.70) | 30.80 (29.80, 31.70) | 0.8 | 30.80 (29.80, 31.80) | 30.80 (29.80, 31.80) | 30.80 (29.70, 31.80) | >0.9 |
| PLT | 242 (206, 280) | 244 (209, 282) | 240 (202, 278) | <0.001 | 244 (208, 282) | 247 (212, 283) | 237 (202, 278) | <0.001 |
| TyG | 1.48 (1.14, 1.85) | 1.42 (1.08, 1.78) | 1.58 (1.24, 1.94) | <0.001 | 1.49 (1.15, 1.85) | 1.43 (1.10, 1.79) | 1.57 (1.24, 1.94) | <0.001 |
| MHR | 0.38 (0.30, 0.49) | 0.37 (0.28, 0.47) | 0.41 (0.32, 0.52) | <0.001 | 0.38 (0.30, 0.48) | 0.36 (0.28, 0.47) | 0.40 (0.32, 0.51) | <0.001 |
| NHR | 2.93 (2.27, 3.73) | 2.80 (2.13, 3.56) | 3.14 (2.47, 3.96) | <0.001 | 2.92 (2.28, 3.73) | 2.80 (2.17, 3.57) | 3.14 (2.46, 3.96) | <0.001 |

^1^ n (%); Median (Q1, Q3)

^2^ Pearson’s Chi-squared test; Wilcoxon rank sum test

BMI: Body Mass Index; SBP: Systolic Blood Pressure, DBP: Diastolic Blood Pressure, ALT: Alanine Aminotransferase; AST: Aspartate Aminotransferase; GGT: Gamma-Glutamyl Transferase; ALP: Alkaline Phosphatase; TBIL: Total Bilirubin; DBIL: Direct Bilirubin; IBIL: Indirect Bilirubin; TP: Total Protein; ALB: Albumin; GLB: Globulin; BUN: Nitrogen; Cr: Creatinine; UA: Uric Acid; EGFR: Estimated Glomerular Filtration Rate; TG: Triglycerides; TC: Total Cholesterol; HDL: High-Density Lipoprotein Cholesterol; LDL: Low-Density Lipoprotein Cholesterol; FBG: Fasting Blood Glucose; WBC: White Blood Cell; NEUT: Neutrophil Count; LYM: Lymphocyte Count; MONO: Monocyte Count; RBC: Red Blood Cell; HGB: Hemoglobin; HCT: Hematocrit; MCH: Mean Corpuscular Hemoglobin; PLT: Platelet Count; TyG: Triglyceride-Glucose; MHR: Monocyte to High-Density Lipoprotein Cholesterol Ratio; NHR: Neutrophil to High-Density Lipoprotein Cholesterol Ratio.

**Table S2.** Baseline Characteristics of the patients in the Completed Follow-Up Group and Lost to Follow-Up Group.

| **Variables** | **Completed Follow-Up Group,**  N = 6,270*^1^* | **Lost to Follow-Up Group‌,**  N = 510*^1^* | **p-value***^2^* |
| --- | --- | --- | --- |
| Gender |  |  | >0.9 |
| Female | 1,929 (31%) | 158 (31%) |  |
| Male | 4,341 (69%) | 352 (69%) |  |
| Age | 56 (47, 65) | 57 (46, 65) | 0.6 |
| Height | 170 (164, 175) | 170 (164, 175) | 0.8 |
| Weight | 74 (66, 83) | 73 (66, 83) | 0.8 |
| BMI | 26.0 (24.0, 28.1) | 26.0 (24.1, 28.0) | 0.7 |
| SBP | 136 (123, 150) | 136 (123, 148) | 0.8 |
| DBP | 81 (74, 90) | 82 (74, 90) | 0.8 |
| ALT | 19 (14, 27) | 19 (14, 27) | 0.6 |
| AST | 20 (17, 23) | 20 (17, 24) | 0.4 |
| ALT/AST | 1.01 (0.80, 1.29) | 1.01 (0.79, 1.33) | >0.9 |
| GGT | 26 (18, 39) | 26 (19, 39) | 0.5 |
| ALP | 65 (55, 76) | 65 (55, 79) | 0.4 |
| TBIL | 11.6 (9.1, 15.1) | 11.4 (9.1, 15.3) | 0.6 |
| DBIL | 4.30 (3.40, 5.40) | 4.20 (3.30, 5.40) | 0.7 |
| IBIL | 7.3 (5.4, 9.8) | 7.1 (5.5, 10.1) | 0.7 |
| TP | 74.6 (72.0, 77.2) | 74.3 (72.2, 77.0) | 0.5 |
| ALB | 47.80 (46.10, 49.50) | 47.70 (46.30, 49.50) | >0.9 |
| GLB | 26.7 (24.6, 28.9) | 26.6 (24.6, 28.9) | 0.7 |
| ALB/GLB | 1.79 (1.63, 1.97) | 1.80 (1.63, 1.96) | 0.7 |
| BUN | 4.90 (4.20, 5.70) | 4.95 (4.20, 5.70) | 0.8 |
| Cr | 75 (65, 84) | 75 (66, 85) | 0.5 |
| UA | 344 (290, 400) | 341 (288, 400) | 0.7 |
| EGFR | 99 (89, 108) | 99 (90, 108) | 0.8 |
| BUN/Cr | 0.066 (0.056, 0.078) | 0.067 (0.056, 0.077) | 0.9 |
| TG | 1.44 (1.03, 2.07) | 1.52 (1.06, 2.14) | 0.2 |
| TC | 4.82 (4.19, 5.47) | 4.76 (4.15, 5.43) | 0.3 |
| HDL | 1.16 (1.02, 1.34) | 1.13 (1.02, 1.36) | 0.6 |
| LDL | 2.81 (2.29, 3.32) | 2.73 (2.24, 3.26) | 0.056 |
| HDL/TC | 0.24 (0.21, 0.29) | 0.25 (0.21, 0.30) | 0.6 |
| FBG | 6.04 (5.78, 6.43) | 6.02 (5.77, 6.38) | 0.5 |
| WBC | 6.22 (5.35, 7.26) | 6.13 (5.32, 7.22) | 0.2 |
| NEUT | 3.41 (2.80, 4.15) | 3.35 (2.72, 4.07) | 0.2 |
| LYM | 2.11 (1.74, 2.55) | 2.12 (1.72, 2.55) | 0.8 |
| MONO | 0.45 (0.37, 0.54) | 0.45 (0.37, 0.55) | 0.8 |
| RBC | 151 (140, 160) | 151 (140, 160) | 0.6 |
| HGB | 4.90 (4.59, 5.20) | 4.88 (4.56, 5.17) | 0.2 |
| HCT | 0.45 (0.43, 0.48) | 0.45 (0.43, 0.48) | 0.6 |
| MCH | 30.70 (29.80, 31.70) | 30.80 (29.80, 31.70) | 0.6 |
| PLT | 242 (206, 280) | 242 (205, 274) | 0.3 |
| TyG | 1.48 (1.14, 1.85) | 1.51 (1.16, 1.89) | 0.2 |
| MHR | 0.38 (0.30, 0.49) | 0.39 (0.30, 0.49) | >0.9 |
| NHR | 2.93 (2.27, 3.73) | 2.87 (2.18, 3.67) | 0.2 |

^1^ n (%); Median (Q1, Q3)

^2^ Pearson’s Chi-squared test; Wilcoxon rank sum test

BMI: Body Mass Index; SBP: Systolic Blood Pressure, DBP: Diastolic Blood Pressure, ALT: Alanine Aminotransferase; AST: Aspartate Aminotransferase; GGT: Gamma-Glutamyl Transferase; ALP: Alkaline Phosphatase; TBIL: Total Bilirubin; DBIL: Direct Bilirubin; IBIL: Indirect Bilirubin; TP: Total Protein; ALB: Albumin; GLB: Globulin; BUN: Nitrogen; Cr: Creatinine; UA: Uric Acid; EGFR: Estimated Glomerular Filtration Rate; TG: Triglycerides; TC: Total Cholesterol; HDL: High-Density Lipoprotein Cholesterol; LDL: Low-Density Lipoprotein Cholesterol; FBG: Fasting Blood Glucose; WBC: White Blood Cell; NEUT: Neutrophil Count; LYM: Lymphocyte Count; MONO: Monocyte Count; RBC: Red Blood Cell; HGB: Hemoglobin; HCT: Hematocrit; MCH: Mean Corpuscular Hemoglobin; PLT: Platelet Count; TyG: Triglyceride-Glucose; MHR: Monocyte to High-Density Lipoprotein Cholesterol Ratio; NHR: Neutrophil to High-Density Lipoprotein Cholesterol Ratio.


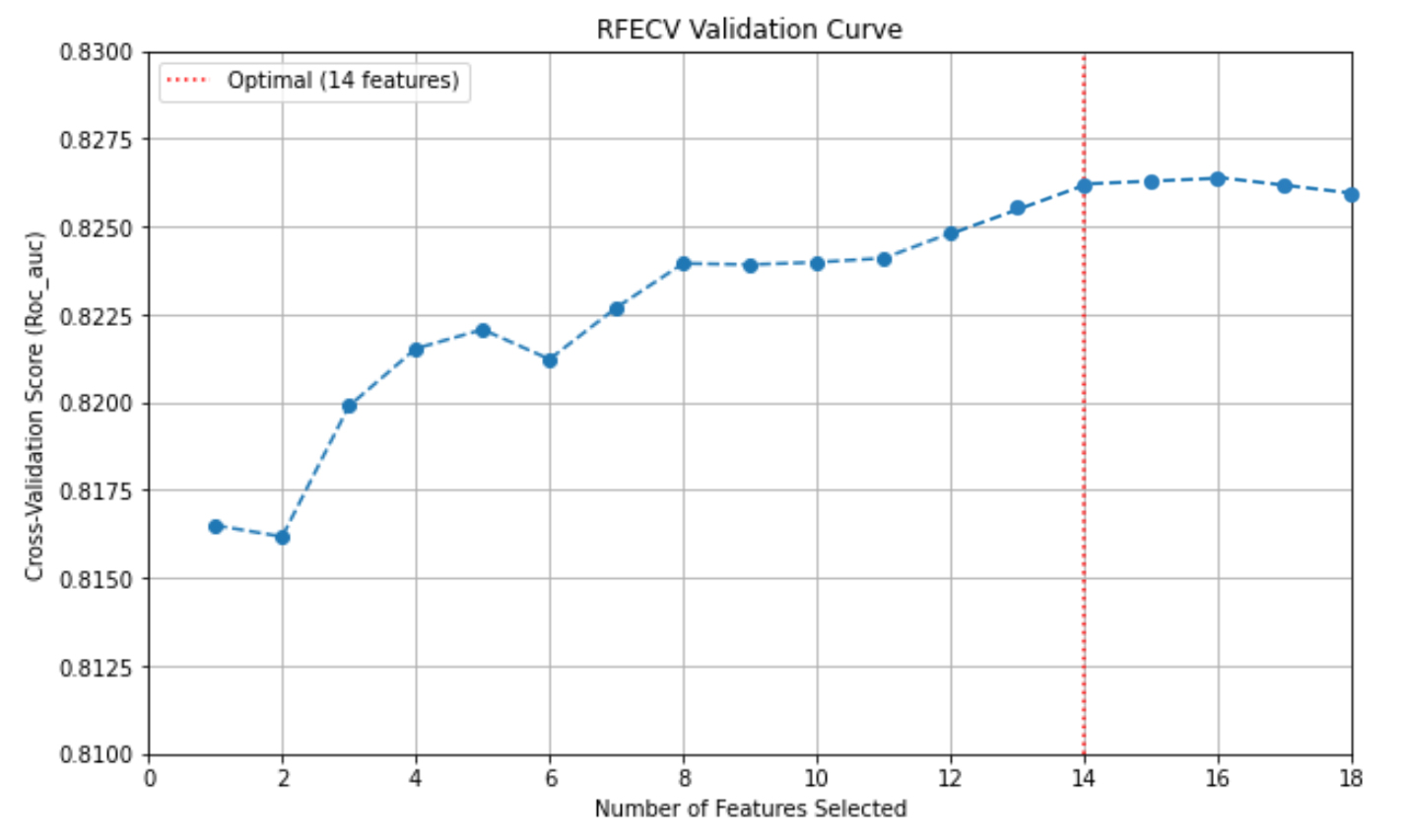


**Figure S1. RFE validation curve with different number of features selected.**

**Table S3. The ordered RFE feature ranking table.**

| **Feature** | **Ranking** |
| --- | --- |
| HCT | 1 |
| HGB | 1 |
| RBC | 1 |
| MONO | 1 |
| FBG | 1 |
| HDL/TC | 1 |
| LDL | 1 |
| HDL | 1 |
| Cr | 1 |
| ALT/AST | 1 |
| Age | 1 |
| Height | 1 |
| Weight | 1 |
| BMI | 1 |
| EGFR | 2 |
| UA | 3 |
| MHR | 4 |
| WBC | 5 |
| MCH | 6 |
| PLT | 7 |
| TyG | 8 |
| NEUT | 9 |
| BUN | 10 |
| NHR | 11 |
| TP | 12 |
| ALP | 13 |
| AST | 14 |
| ALT | 15 |
| SBP | 16 |
| DBP | 17 |
| ALB/GLB | 18 |
| BUN/Cr | 19 |
| GLB | 20 |
| TBIL | 21 |
| DBIL | 22 |
| GGT | 23 |
| TG | 24 |
| TC | 25 |
| Gender | 26 |
| LYM | 27 |
| ALB | 28 |
| IBIL | 29 |

BMI: Body Mass Index; SBP: Systolic Blood Pressure, DBP: Diastolic Blood Pressure, ALT: Alanine Aminotransferase; AST: Aspartate Aminotransferase; GGT: Gamma-Glutamyl Transferase; ALP: Alkaline Phosphatase; TBIL: Total Bilirubin; DBIL: Direct Bilirubin; IBIL: Indirect Bilirubin; TP: Total Protein; ALB: Albumin; GLB: Globulin; BUN: Nitrogen; Cr: Creatinine; UA: Uric Acid; EGFR: Estimated Glomerular Filtration Rate; TG: Triglycerides; TC: Total Cholesterol; HDL: High-Density Lipoprotein Cholesterol; LDL: Low-Density Lipoprotein Cholesterol; FBG: Fasting Blood Glucose; WBC: White Blood Cell; NEUT: Neutrophil Count; LYM: Lymphocyte Count; MONO: Monocyte Count; RBC: Red Blood Cell; HGB: Hemoglobin; HCT: Hematocrit; MCH: Mean Corpuscular Hemoglobin; PLT: Platelet Count; TyG: Triglyceride-Glucose; MHR: Monocyte to High-Density Lipoprotein Cholesterol Ratio; NHR: Neutrophil to High-Density Lipoprotein Cholesterol Ratio.


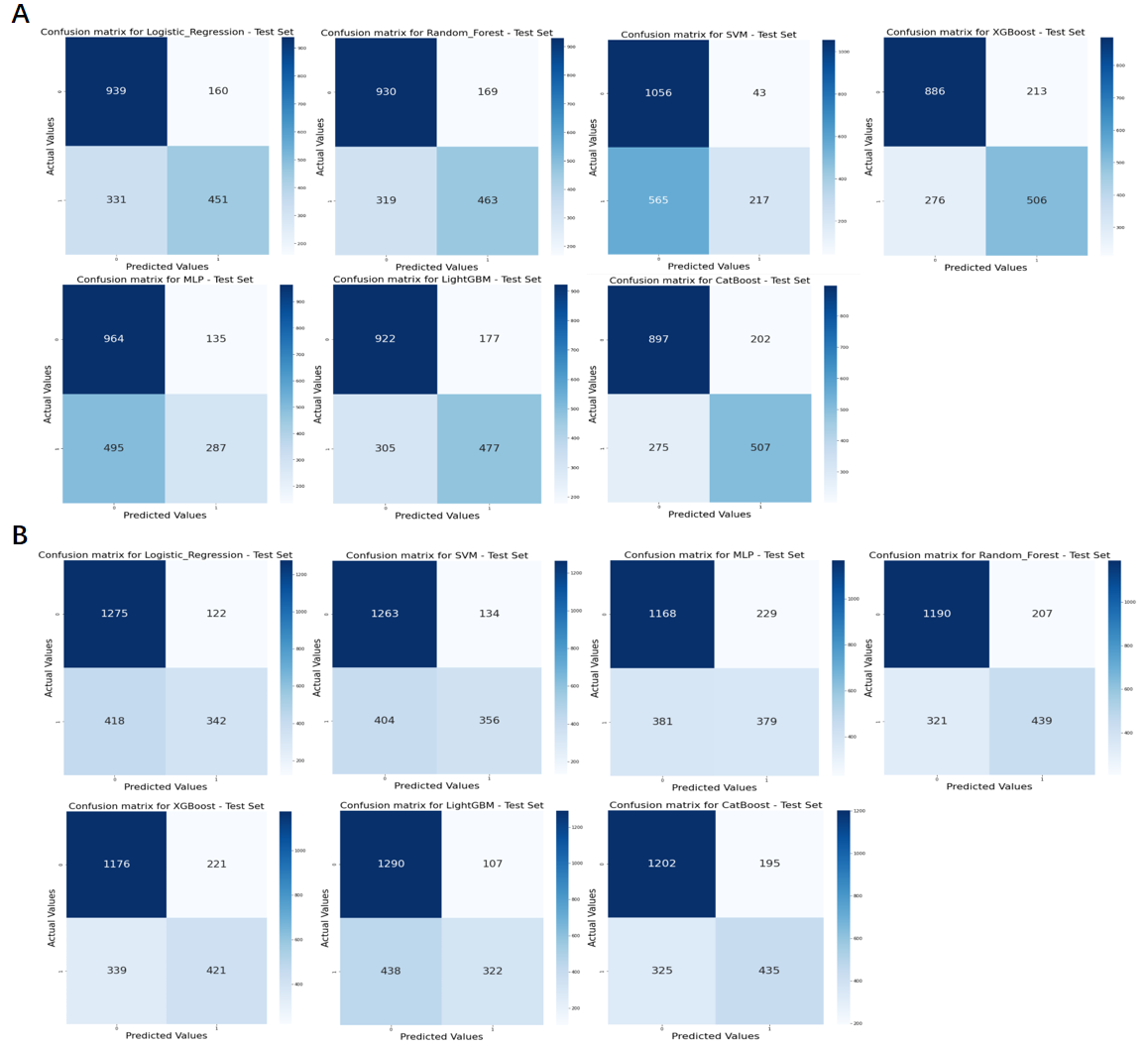


**Figure S2.** The confusion matrix of the 7 models on the test set and the external test set. Plot A depicts the results on the test set, and plot B presents the results on the external test set. SVM: support vector machine; XGBoost: extreme gradient boosting machine; MLP: multiplayer perceptron; LightGBM: light gradient boosting machine; CatBoost: categorical boosting.


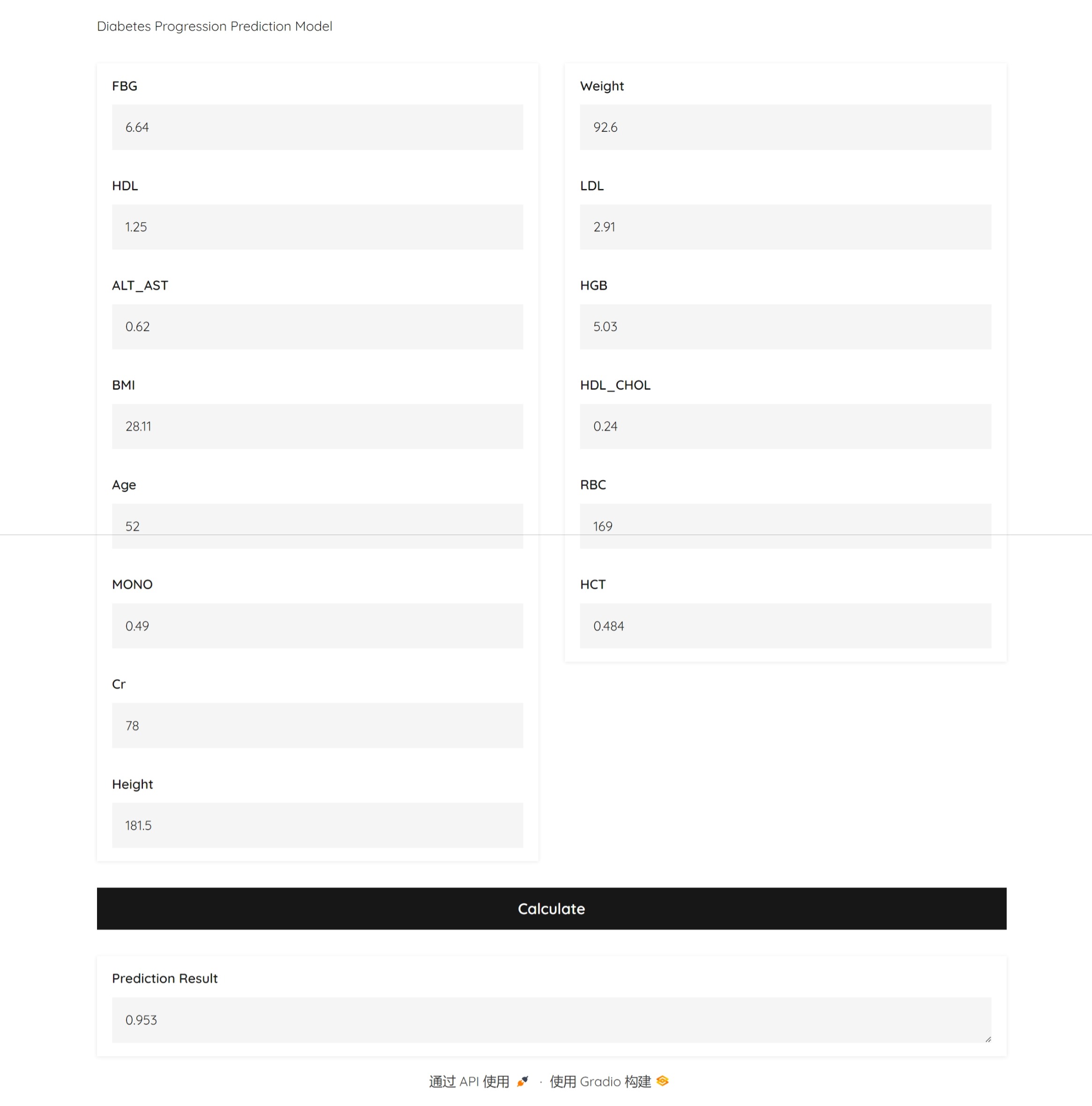


**Figure S3.** The interactive interface developed using Gradio.

The code for this interface is uploaded at https://huggingface.co/zysnathan/diabetes_progression.
